# Supplementary material for: Class-A penicillin binding proteins do not contribute to cell shape but repair cell-wall defects
Source: eLife. 2020 Jan 6;9:e51998. doi: 10.7554/eLife.51998 (PMC7002073; doi:10.7554/eLife.51998)
Supplement: Transparent reporting form [file elife-51998-transrepform.docx]

***eLife’s* transparent reporting form**

We encourage authors to provide detailed information *within their submission* to facilitate the interpretation and replication of experiments. Authors can upload supporting documentation to indicate the use of appropriate reporting guidelines for health-related research (see [EQUATOR Network](http://www.equator-network.org/%20)), life science research (see the [BioSharing Information Resource](https://biosharing.org/)), or the [ARRIVE guidelines](http://www.plosbiology.org/article/info:doi/10.1371/journal.pbio.1000412) for reporting work involving animal research. Where applicable, authors should refer to any relevant reporting standards documents in this form.

If you have any questions, please consult our Journal Policies and/or contact us: [editorial@elifesciences.org](mailto:editorial@elifesciences.org).

**Sample-size estimation**

- You should state whether an appropriate sample size was computed when the study was being designed
- You should state the statistical method of sample size computation and any required assumptions
- If no explicit power analysis was used, you should describe how you decided what sample (replicate) size (number) to use

Please outline where this information can be found within the submission (e.g., sections or figure legends), or explain why this information doesn’t apply to your submission:

For measurements of growth, cell morphology, osmotic shock survival, we typically had 3 independent biological replicates. For every replicate of single-cell measurements we imaged as many cells or single-molecule tracks as possible, within the limited lifespan of good-quality microscopy. Detailed numbers are indicated in the manuscript or methods.

Specifically:

- For all measurements of morphology and fluorescence intensity, we imaged enough cells to have at least 300 cells remaining per replicate after filtering for focus and cell-to-cell proximity.

- For measurements of cell elasticity, about 30 cells were imaged per condition in one experiment. As all conditions were imaged at once, experimental error affects all cells equally.

- For single-molecule tracking, we acquired at least 3 fields of view with hundreds of cells per replicate, giving typically >5000 tracks.

For cell-wall insertion rate, some conditions have only 2 replicates due to constraints on equipment capacity for experiments involving radioactivity.

**Replicates**

- You should report how often each experiment was performed
- You should include a definition of biological versus technical replication
- The data obtained should be provided and sufficient information should be provided to indicate the number of independent biological and/or technical replicates
- If you encountered any outliers, you should describe how these were handled
- Criteria for exclusion/inclusion of data should be clearly stated
- High-throughput sequence data should be uploaded before submission, with a private link for reviewers provided (these are available from both GEO and ArrayExpress)

Please outline where this information can be found within the submission (e.g., sections or figure legends), or explain why this information doesn’t apply to your submission:

Whenever multiple replicates are available, we plotted the individual data points on the same figure.

Detailed source data can be found in the individual excel file for the corresponding figure. See also a separate pdf document for a brief description of the datasets.

We did not have to exclude outliers.

**Statistical reporting**

- Statistical analysis methods should be described and justified
- Raw data should be presented in figures whenever informative to do so (typically when N per group is less than 10)
- For each experiment, you should identify the statistical tests used, exact values of N, definitions of center, methods of multiple test correction, and dispersion and precision measures (e.g., mean, median, SD, SEM, confidence intervals; and, for the major substantive results, a measure of effect size (e.g., Pearson's r, Cohen's d)
- Report exact p-values wherever possible alongside the summary statistics and 95% confidence intervals. These should be reported for all key questions and not only when the p-value is less than 0.05.

Please outline where this information can be found within the submission (e.g., sections or figure legends), or explain why this information doesn’t apply to your submission:

Statistical analysis methods are described in the Methods.

Results from individual replicates are indicated in figures unless noted otherwise. Data points indicated in figures are also given in supplemental datasets.

p-values from t-tests between different conditions are reported in the main text or in the figure. All the tests we report remain significant after Bonferroni’s correction for multiple testing.

(For large datasets, or papers with a very large number of statistical tests, you may upload a single table file with tests, Ns, etc., with reference to sections in the manuscript.)

**Group allocation**

- Indicate how samples were allocated into experimental groups (in the case of clinical studies, please specify allocation to treatment method); if randomization was used, please also state if restricted randomization was applied
- Indicate if masking was used during group allocation, data collection and/or data analysis

Please outline where this information can be found within the submission (e.g., sections or figure legends), or explain why this information doesn’t apply to your submission:

**Additional data files (“source data”)**

- We encourage you to upload relevant additional data files, such as numerical data that are represented as a graph in a figure, or as a summary table
- Where provided, these should be in the most useful format, and they can be uploaded as “Source data” files linked to a main figure or table
- Include model definition files including the full list of parameters used
- Include code used for data analysis (e.g., R, MatLab)
- Avoid stating that data files are “available upon request”

Please indicate the figures or tables for which source data files have been provided:

Detailed source data is provided as additional excel files for each figure. A caption for every dataset is found in a supplementary text document ‘Datasets_Captions.pdf’.
